# Evaluating maternal self -efficacy in managing child obesity-related behaviors: A cross-sectional study

**DOI:** 10.34172/hpp.025.44757

**Published:** 2025-12-30

**Authors:** Mina Hashemiparast, Sousan Mohammad Nejad, Khadijeh Hajimiri

**Affiliations:** ^1^Department of Health Education and Promotion, School of Public Health, Zanjan University of Medical Sciences, Zanjan, Iran; ^2^Social Determinants of Health Research Center, Health and Metabolic Disease Research Institute, Zanjan University of Medical Sciences, Zanjan, Iran

**Keywords:** Behavior, Childhood obesity, Life style, Mothers, Self-efficacy

## Abstract

**Background:**

Maternal self-efficacy is a key determinant in managing behaviors linked to childhood obesity. This study investigated maternal self-efficacy in addressing childhood obesity-related behaviors in Zanjan, Iran.

**Methods:**

This cross-sectional study, conducted in 2023, investigated maternal self-efficacy among 458 mothers of children aged 6–12 years. Participants were selected via cluster sampling from five comprehensive health centers across the city. Data were collected using the validated Persian version of the Children’s Lifestyle Behavior Checklist, which assesses parental perceptions of obesity-related behaviors and self-efficacy in addressing them. Independent t-tests and multiple linear regression analyses were conducted using SPSS (version 23), with statistical significance set at *P*<0.05.

**Results:**

Most mothers (96.9%, 95% CI: 94.8% to 98.2%) reported difficulties in managing obesity-related behaviors, with 36.9% (95% CI: 32.4% to 41.6%) showing low self-efficacy, particularly for sedentary lifestyles. Significant differences in problem perception (*P*<0.001, 95% CI: –64.83 to –30.54) and self-efficacy (*P*<0.001, 95% CI: 14.51 to 27.66) were observed between mothers of normal-weight versus overweight/obese children. Regression analysis indicated that maternal education (β=0.202, 95% CI: 6.910 to 20.388, *P*=0.0001) and child BMI (β=-0.303, 95% CI: -3.343 to -1.812, *P*=0.0001) were key predictors of self-efficacy.

**Conclusion:**

Sedentary lifestyles were a major concern among mothers. They reported low confidence in managing obesity-related behaviors, underscoring the need for targeted interventions. Specifically, evidence-based educational programs are essential to enhance mothers’ capacity to regulate children’s screen time and promote healthy lifestyle behaviors.

## Introduction

 Childhood obesity has emerged as rapidly intensifying global public health issue, often described as a “silent epidemic”. Although the prevalence of this varies considerably across nations and within regions, the global trajectory continues to ascend, reflecting a widespread and persistent trend.^1,2^ Within-country disparities largely reflect socioeconomic status. In low- and middle-income countries, children from higher-income families show higher rates of overweight and obesity, whereas in high-income nations, pediatric obesity is more common among socioeconomically disadvantaged children.^3^ Obesity harms child development by increasing cardiovascular, metabolic, and psychosocial risks, and often persists into adulthood, raising the likelihood of later morbidity and mortality.^4^

 A 2000–2023 meta-analysis found global obesity, overweight, and excess weight rates of 8.5%, 14.8%, and 22.2% among youths, meaning roughly one in five is affected. Prevalence differs by regional income and the Human Development Index. Its causes are multifactorial, spanning genetic, behavioral, environmental, and socio-cultural factors, and necessitate targeted interventions.^2^ In Iran, meta-analytic findings show that 12.43% of children and adolescents are overweight and 6.51% are obese, highlighting the regional burden of this public health challenge.^5^

 Childhood obesity often persists into adulthood and poses significant public health challenges, making prevention a critical priority.^6^ Systematic reviews demonstrate that successful childhood obesity interventions are multifaceted and largely family-centered, addressing diet, physical activity, and behavioral changes.^7,8^ An umbrella review of Cochrane studies emphasizes the key role of parental involvement and role modeling in children’s weight management.^9^ Accordingly, interventions often target parents, but many still struggle to address obesity-related behaviors, highlighting the need for tailored support.^4,10,11^

 Childhood obesity is a complex and multifactorial condition shaped by biological, social, parental, and psychological factors.^12^ Among the psychological factors, self-efficacy plays a key role. It reflects an individual’s belief in their capacity to manage future situations.^13^ In this study, maternal self-efficacy refers to mothers’ confidence in managing their children’s obesity-related behaviors such as eating habits, physical activity, screen time, and weight management as measured by the problem and confidence scales.^14^ Parental behaviors, especially maternal lifestyle choices, strongly shape children’s health habits. Maternal diet, physical activity, and health awareness are central to promoting healthy behaviors and preventing childhood obesity.^15^ Parents, particularly mothers, with high self-efficacy are more likely to promote healthy lifestyles by improving diet, increasing physical activity, and modeling positive behaviors. Evidence shows that interventions grounded in Social Cognitive Theory can enhance children’s dietary habits, reduce screen time, and lower body mass index (BMI), especially among low-income families.^16^ Similarly, a study in low-income parent-preschooler dyads showed improvements in children’s dietary habits, food security, BMI, and blood pressure, along with increased parental nutrition knowledge, self-efficacy, and healthier home eating practices.^17^ Furthermore, Alexandrou et al’s^18^ m-Health intervention improved children’s dietary habits and physical activity, reduced screen time and intake of sugary foods, and enhanced parents’ self-efficacy in promoting healthy lifestyles. Rajan et al^19^ demonstrated that maternal self-efficacy acts as a protective factor against higher BMI in Mexican origin children with negative temperament.

 Identifying early challenges in parental management of children’s lifestyle behaviors and their self-efficacy is essential for fostering healthy development and mitigating future health risks.^20,21^ Evaluating children’s lifestyles behaviors supports the development of preventive strategies against rising lifestyle-related diseases. This study, examined mothers’ self-efficacy in managing behaviors associated with childhood overweight and obesity using the Lifestyle Behavior Checklist (LBC), a reliable tool that assesses parents’ perceptions of obesity-related behaviors such as diet, physical activity, and screen time and their confidence in managing them.^14^ The tool has demonstrated reliability and has been successfully applied in Iran as well as in other countries.^4,14,22,23^

## Methods

###  Study design and sampling

 This cross-sectional study was conducted in 2023 among 458 mothers with children aged 6 to 12 years in Zanjan, Iran. The inclusion criteria for participants were as follows: (1) mothers with at least one child aged 6 to 12 years; (2) children with medical records at local health facilities in Zanjan; (3) children without documented severe mental or physical disorders; and (4) mothers who provided informed consent to participate in the study.

 The target population comprised 63,078 mothers with children aged 6 to 12 years. The initial sample size was calculated using the standard formula for estimating proportions in large populations, with a 95% confidence level (Z = 1.96), an anticipated proportion of 0.50 for maternal self-efficacy, and a margin of error set at 0.05. Employing a proportion of 0.50, which represents the point of maximum variability, allowed for a conservative and reliable estimation of the required sample size. Consequently, the preliminary sample size was determined to be 381 participants. To accommodate the cluster sampling framework, which included five urban health centers, and to adjust for the inflation in variance arising from intra-cluster correlation, a design effect (DEFF) of 1.2 was incorporated into the sample size calculation. This methodological refinement increased the final sample size to 458 participants, enhancing the precision and generalizability of the study estimates. Although the initial sample size was calculated for estimating a single proportion, the final sample of 458 participants was more than adequate for the multivariable regression analyses. Based on the recommended rule of 10–15 participants per predictor, the three predictors included in the final models were well supported, ensuring sufficient statistical power.

 Among the 18 comprehensive health service centers in Zanjan, five were randomly selected to represent the northern, southern, central, eastern, and western areas of the city. This regional stratification aimed to balance representativeness with operational feasibility. Within each selected center, eligible participants were identified through simple random sampling. Lists of mothers meeting the inclusion criteria were extracted from electronic health records and randomized using Microsoft Excel. To maintain proportionality across centers, quota sampling based on proportional allocation was used, and participant recruitment continued at each center until the assigned quota was fulfilled.

###  Instruments

 A self-administered structured questionnaire was used to collect data, comprising two main sections. The first section gathered demographic information, including parental age, education level, occupation, and family structure. Child characteristics, such as date of birth, gender, height, and weight, were obtained from health records. Children’s BMI was calculated and converted into BMI Z-scores, categorized according to the World Health Organization (WHO) 2007 guidelines as follows: healthy weight (BMI Z-score between -2 and + 1), overweight (BMI Z-score ≥ 1 to + 2), or obese (BMI Z-score ≥ + 2).^24^ Children classified as underweight were excluded from the study.

 The second section utilized the Persian version of the Children’s LBC, validated by Omidvar et al^4^ with reported Cronbach’s alpha values of 0.80 for the Problem scale and 0.95 for the Confidence scale. Spearman’s correlation coefficients further confirmed the reliability of these scales, with values of 0.74 and 0.70 for the Problem and Confidence scales, respectively. In the original version of the instrument, sedentary behaviors were included as a subcomponent of the physical activity scale, which comprised five dimensions overall.^23^ However, in the Iranian adaptation, sedentary behaviors and physical activity were validated as two distinct dimensions, reflecting a culturally and contextually sensitive revision of the instrument’s structure.

 The LBC consists of 25 items divided into two subscales: (a) Problem scale and (b) Confidence scale. The Problem scale assesses parents’ perceptions of problematic behaviors related to childhood obesity, covering six domains: food-related problem behaviors (7 items), fussy eating (4 items), overeating behaviors (4 items), low interest in physical activity (3 items), poor self-image (5 items), and sedentary behaviors (2 items). Mothers rated each behavior on a scale from 1 (not at all problematic) to 7 (very problematic), yielding a score range of 25 to 175, with a cut-off point above 50 indicating problematic behavior. The Confidence scale evaluates parents’ self-efficacy in managing these behaviors, rated from 1 (certainly cannot manage) to 10 (certainly can manage), with scores ranging from 25 to 250 and a cut-off point below 204.^14^ For behaviors not observed, parents were asked to provide hypothetical self-efficacy ratings. The internal consistency of the Persian LBC was re-examined in a pilot sample of 30 participants using Cronbach’s alpha (Problem scale: α = 0.847; Confidence scale: α = 0.978), since the instrument had already undergone full psychometric validation in a previous Iranian study.^4^

###  Data collection and analysis

 Data collection was conducted with voluntary participants at each health center following scheduled appointments for questionnaire completion. The researcher provided a clear explanation of the study’s objectives, and informed consent was obtained from all participants prior to data collection. Participants completed the questionnaire in approximately 10–15 minutes. To ensure data completeness and enhance the reliability of statistical analyses, the questionnaires were carefully administered to minimize missing responses. For variables exhibiting less than 5% missing data, imputation was conducted using the nearest median method. This technique involves replacing missing values with the median value from the most similar cases within the dataset, identified based on a selected distance metric.

###  Statistical analysis

 Statistical analyses were performed using IBM SPSS Statistics for Windows, version 23.0 (IBM Corp., Armonk, NY, USA). Categorical variables were summarized using frequencies and percentages, while numerical variables were described using means, standard deviations (SD), and minimum and maximum values. Independent t-tests were used to compare mean scores. Two separate multiple linear regression models were conducted to examine the associations of predictors (maternal education, BMI, number of children, child age, and BMI) with mothers’ perceptions of obesity-related problematic behaviors and their self-efficacy in managing these behaviors. Categorical predictors were dummy-coded (0 and 1). Univariable linear regressions were first performed, and variables with *P* < 0.05, along with theoretically important predictors identified in prior literature, were included in the multivariable models using the Enter method. Multicollinearity was assessed using Variance Inflation Factors, all of which were below the recommended threshold. Regression assumptions—including normality, homoscedasticity, independence of residuals, and the absence of influential outliers—were examined through standard diagnostic tests and plots, and no major violations were observed. Normality of the data was further supported by acceptable skewness and kurtosis values (skewness < 2, kurtosis < 7), consistent with the criteria proposed by Kim.^25^ Statistical significance was set at *P* < 0.05.

## Results

 A total of 458 biological mothers of children aged 6–12 years participated in this study. Participant characteristics are presented in Table 1. Among the children, 25.5% % were classified as obese.

**Table 1:** General characteristics of participants' in this study (n = 458)

**Variable**	**Mean**	**SD**
Mother age (year)	37.12	5.43
Father age (year)	41.01	5.47
Child age (year)	7.69	1.89
	**Number**	**%**
Child’s gender		
Girl	222	48.5
Boy	236	51.5
Child weight status		
Normal ( > − 2 and < + 1)	280	61.1
Overweight ( + 1 to < + 2)	61	13.3
Obese ( + 2 to < + 3)	117	25.5
Mothers’ education		
High school and diploma	230	50.2
University degree	228	49.8
Fathers’ education		
High school and diploma	239	52.2
University degree	228	47.8
Mothers’ job status		
Housewife	355	77.5
Employed	103	22.5
Fathers’ job status		
Employee	163	35.6
Worker	62	13.5
Self-employed	212	46.3
Other	21	4.6
Family structure		
Both parents	452	98.7
Only mother	6	1.3

 Based on established clinical threshold values specifically, a score above 50 on the behavior problems scale and below 204 on the confidence scale^14^ this study found that only 3.1% of participating mothers scored below 50 on the behavior problems scale. This finding indicates that the majority of mothers perceive childhood obesity-related behaviors as problematic and concerning. Furthermore, 36.9% of mothers scored above 204 on the self-efficacy scale, reflecting a high level of confidence in their ability to manage these behaviors effectively. The result corresponds to the culturally defined role of Iranian mothers as primary caretakers and regulators of children’s health behaviors, underscoring their considerable awareness and concern about these health challenges. As shown in Table 2, the highest mean problem score and the lowest mean confidence score were observed in the sedentary behavior domain.

**Table 2:** Scores for the dimensions of the Lifestyle Behavior Checklist (LBC) (n = 458)

	**Problem score Possible range**	**Problem score**		**Problem score out of 100**		**Confidence score Possible range**	**Confidence score**		**Confidence score out of 100**	
		**Mean**	**SD**	**Mean**	**SD**		**Mean**	**SD**	**Mean**	**SD**
FE	4-28	10.18	5.83	25.76	24.32	4-40	29.43	7.01	70.63	19.47
FPB	7-49	17.15	7.14	24.16	17.02	7-70	52.92	11.21	72.89	17.79
OB	4-28	11.54	5.00	31.41	20.86	4-40	28.72	6.91	68.68	19.20
LPA	3-21	6.24	4.08	18.00	22.68	3-30	23.40	5.74	75.55	21.27
PSI	5-35	9.13	5.83	13.76	19.44	5-50	40.79	8.83	79.53	19.62
SB	2-14	7.84	3.15	48.67	26.28	2-20	13.51	3.88	63.94	21.56
Total score	25-175	161.74	88.77	-	-	25-250	188.74	36.35	-	-
Problem score above than 50(N, %)		444	96.9				(95% CI: –64.83 to –30.54)			
Confidence score above than 204 (N, %)		169	36.9				(95% CI: 14.51 to 27.66)			

FE, Fussy eating; FPB, Food-related problem behaviors; OB, Overeating behaviors; LPA, Low interest in physical activity; PSI, Poor self-image; SB, sedentary behaviors.

 Mean scores on the problem and confidence scales, along with their respective items, were compared between mothers of children with normal weight and those with overweight or obese children (Table 3). Results from an independent t-test indicated that mothers of overweight or obese children had a significantly higher mean problem scale score than mothers of normal-weight children (t = -5.49; df = 306.82; *P* < 0.0001; Cohen’s d = 0.542). Additionally, mothers of overweight or obese children reported a significantly lower mean self-efficacy score (t = 6.309; df = 456; *P* < 0.0001; Cohen’s d = 0.599).

**Table 3:** Group means for the Lifestyle Behavior Checklist (LBC) items based on child weight status (n = 458)

	**Problem scale**								**Confidence scale**							
	**Healthy weight (** ***n*** **=280)**		**Overweigh/ obese (** ***n*** **=178)**		***t***	***df***	***P *** **value**	**Effect sizes (Cohen's d)**	**Healthy weight (** ***n*** **=280)**		**Overweigh/ obese (** ***n*** **=178)**		***t***	***df***	***P *** **value**	**Effect sizes (Cohen's d)**
	**Mean**	**SD**	**Mean**	**SD**					**Mean**	**SD**	**Mean**	**SD**				
FE	10.34	5.78	9.93	5.92	0.73	456	0.410	0.07	30.17	6.89	28.26	7.05	2.86	456	0.0004^*^	0.27
FPB	15.69	5.79	19.45	8.39	-5.29	282	< 0.0001^*^	0.52	55.34	10.13	49.11	11.79	5.79	333.28	< 0.0001^*^	0.56
OB	9.74	3.71	14.37	5.46	-9.92	280.95	< 0.0001^*^	0.44	30.35	6.70	26.16	6.46	6.61	456	< 0.0001^*^	0.63
LPA	5.83	3.85	6.89	4.35	-2.67	-2.67	0.008*	0.20	24.49	5.37	21.68	5.89	5.25	455	< 0.0001^*^	0.49
PSI	8.35	5.18	10.36	6.56	-3.45	311.59	< 0.0001^*^	0.34	42.62	8.12	37.90	9.15	5.60	341.54	< 0.0001^*^	0.54
SB	7.44	2.99	8.48	3.29	-3.48	456	< 0.0001^*^	0.33	14.01	3.76	12.72	3.93	3.49	455	< 0.0001^*^	0.33
Total Problem scale	143.20	76.03	191.21	99.36	-5.49	306.82	< 0.0001^*^	0.54								
Total Confidence scale									196.95	33.63	175.75	36.99	6.30	456	< 0.0001^*^	0.59

FE, Fussy eating; FPB, Food-related problem behaviors; OB, Overeating behaviors; LPA, Low interest in physical activity; PSI, Poor self-image; SB, sedentary behaviors.

 Multiple linear regression analyses were conducted to examine the relationship between demographic variables and mothers’ perceptions of obesity-related behavioral issues in children, as well as their confidence in addressing these behaviors (Table 4). After controlling for confounders, a one-unit increase in child BMI was associated with a significant 0.324-unit increase in mothers’ perception scores for obesity-related behavioral issues. Mothers with a university education exhibited significantly lower perception scores (0.169 units) compared to those with a high school diploma or less. This model accounted for 11.6% of the variance in perception scores. Similarly, a one-unit increase in child BMI was associated with a significant 0.331 -unit decrease in mothers’ confidence scores for managing obesity-related behaviors, after adjusting for covariates. Mothers with a university education reported significantly higher confidence scores (0.192 units) than those with lower educational attainment. The proposed model explained 13.3% of the variance in maternal self-efficacy scores, primarily attributed to demographic characteristics and child BMI.

**Table 4:** Assessment of the relationship between Lifestyle Behavior Checklist (LBC) score and demographic variables

**Predictor**	**B**	**SE**	**β**	**t(df=441)**	***P***	**95% CI (Lower–Upper)**	**Tolerance**	**VIF**
Child’s BMI	–2.798	0.380	–0.331	–7.366	< 0.001	–3.544 to –2.051	0.972	1.029
Mothers’ BMI	2.034	2.099	0.044	0.969	0.333	–2.090 to 6.159	0.963	1.039
Mothers’ education	12.701	2.962	0.192	4.288	< 0.001	6.880 to 18.522	0.977	1.023
R = 0.365		R^2^ = 0.133		ADJ.R^2^ = 0.127		Durbin-Watson = 1.76		
Dependent variable: Confidence total score								
**Predictor**	**B**	**SE**	**β**	**t(df=441)**	***P***	**95% CI (Lower–Upper)**	**Tolerance**	**VIF**
Child’s age	-2.366	2.213	-0.052	-1.069	0.286	-6.714 to1.983	0.859	1.164
Child’s BMI	7.190	1.076	0.324	6.680	< 0.001	5.075 to 9.306	0.854	1.170
Mothers’ education	-29.332	7.802	-0.169	-3.760	< 0.001	-44.665 to -13.999	0.995	1.006
R = 0.340		R^2^ = 0.116		ADJ.R^2^ = 0.110		Durbin-Watson = 1.41		
Dependent variable: Problem total score								

B, Unstandardized coefficients; SE, Standard error; β, Standardized coefficients; CI, Confidence interval; VIF, Variance inflation factor.

## Discussion

 In this study, 25.5% of children were classified as obese based on their health records, a finding consistent with Omidvar et al,^4^ who reported a similar prevalence of overweight or obese children. A 2022 systematic review by Saeidi et al^26^ found that among primary school students in Iran, the prevalence of obesity and overweight was 14.3% and 18.8%, respectively.

 The current study revealed that 96.9% of mothers scored above 50 on the Problem Scale, indicating significant concerns about obesity-related behaviors in their children. However, only 36.9% of mothers demonstrated high self-efficacy in managing these behaviors, as measured by the Confidence Scale. It has been observed that a mother’s self-efficacy in managing her child’s weight is associated with how she perceives her child’s weight status and her approach to addressing related challenges. Conversely, low parental self-efficacy may represent a barrier for parents attempting to modify their children’s eating habits and physical activity behaviors.^27^ However, this confidence should be interpreted with caution considering socio-cultural challenges prevalent in Iran, including limited access to structured educational programs on child nutrition and physical activity, social norms affecting maternal roles, and variability in health literacy. These findings highlight the critical need for culturally tailored interventions aimed at empowering mothers through education, resources, and supportive policies to effectively address childhood obesity in Iran. Furthermore, future research should prioritize identifying barriers to maternal self-efficacy and devising community-based approaches aimed at enhancing mothers’ capabilities in managing obesity-related behaviors in children.

 Building on the assessment of mothers’ perceptions of childhood obesity-related problems and their self-efficacy, the next step involves identifying and addressing the primary challenges they encounter in managing these behaviors. Among the identified challenges, sedentary behaviors particularly excessive television viewing and video or computer gaming were the most prevalent, with mothers reporting the lowest confidence in addressing these issues. These findings align with prior studies,^4,14,22^ which highlight the widespread difficulty of regulating children’s screen time, potentially due to limited access to alternative activities or parental support resources. The pervasive use of mobile devices has further integrated online gaming into children’s daily routines, significantly increasing average gaming time.^28^ Members of Generation Alpha, often described as digital natives, typically begin using digital devices as early as kindergarten, rapidly developing proficiency in internet use. Many are introduced to tablets by age two, with “tablet” occasionally being among their first words. However, this early proficiency in gaming may contribute to behavioral challenges, including aggression.^29^ Excessive screen time is also associated with an increased risk of obesity in children and adolescents. Research indicates that individuals spending more than one hour daily on television or video games are more likely to be overweight or obese, particularly when physical activity guidelines are not met.^30-32^ According to Bandura’s social cognitive theory, parents significantly influence their children’s screen-related behaviors through role modeling, rule-setting, and shaping the social environment. Parental beliefs, attitudes, and norms form a key context for children’s behavioral development. Although scientific evidence offers strategies for managing screen time, inconsistent guidelines may cause confusion among parents.^33^ Parents with higher levels of self-efficacy are more likely to implement consistent and effective parenting strategies that encourage healthy screen-related behaviors in children. In contrast, those with lower self-efficacy may struggle to establish or maintain screen time rules and are more prone to discontinue their efforts when encountering obstacles.^34^

 The study demonstrated that mothers ranked children’s overeating as the second most challenging behavior to manage and reported limited confidence in addressing it effectively. Specifically, they identified rapid eating, excessive food consumption, resistance to food refusal, and frequent demands for additional servings as significant challenges, expressing uncertainty about their ability to manage these behaviors successfully. Similarly, a study involving children with severe early-onset obesity identified overeating, requests for larger portions, consumption of unhealthy snacks, frequent food requests between meals, and rapid eating as the most prevalent problematic eating behaviors. Participants in this study reported the lowest confidence in managing overeating, rapid eating, unhealthy snacking, requests for larger portions, and complaints related to being overweight.^35^

 Although cultural differences exist across countries, studies using the Lifestyle Behavior Checklist have consistently identified similar patterns. For instance, research involving Mexican mothers of children with overweight or obesity reported that excessive television viewing and rapid eating were the most challenging behavioral issues related to their children’s lifestyle.^36^ Comparable findings were reported among Australian and Swedish mothers, who highlighted excessive eating and excessive television viewing as the primary behavioral concerns.^14,23^ In evaluations of confidence, Mexican mothers reported the lowest confidence in managing their children’s frequent requests for additional meals,^36^ whereas Australian and Swedish mothers indicated the least confidence in addressing their children’s excessive eating habits.^14,23^ A critical factor in this context is the adverse effect of low caregiver confidence on child behavior. Reduced caregiver self-efficacy exacerbates behavioral challenges, creating a negative feedback loop wherein problematic behaviors diminish caregiver confidence, leading to feelings of inadequacy, difficulties in child management, and further intensification of behavioral issues.^35^ Emotional overeating in children may be a learned behavior shaped by parental modeling and emotion-based feeding practices. Such strategies, though often intended to soothe or comfort, may inadvertently hinder the development of children’s self-regulation.^27^

 Mothers of children with overweight or obesity reported significantly higher scores on obesity-related problematic behaviors and lower self-efficacy in managing these behaviors compared to mothers of children with normal weight, as indicated by total scale scores. This finding contrasts with Omidvar et al,^4^ who reported no correlation between child BMI and these scores. This discrepancy may be partly attributed to the narrower age range examined in our study (6–12 years) compared to the broader range used by Omidvar et al (3–12 years). Additionally, cultural differences in parenting styles may account for further variation. It is also worth noting that the inclusion of fathers in their study may have influenced the reported outcomes.^4^ Similarly, Ek et al^14^ observed higher scores on the problem behavior scale among mothers of overweight or obese children in Sweden, though they found no significant differences in self-efficacy. In contrast, West and Sanders^23^ reported significant differences in both problem behavior and confidence scales between mothers of normal-weight and overweight children. Studies conducted in Brazil^36^ and Australia^23^ further support these findings, indicating that mothers of overweight or obese children reported more lifestyle-related behavioral issues and lower self-efficacy compared to mothers of children with healthy weight. However, research in the Netherlands^22^ and Sweden^14^ found that while mothers of overweight or obese children reported elevated lifestyle-related behavioral problems, their self-efficacy did not differ significantly from that of mothers of normal-weight children.

 In this study, no significant differences were observed in Fussy eating behaviors, such as whining, yelling, or tantrums, between mothers of normal-weight and overweight children. This finding contrasts with Omidvar et al,^4^ who reported a higher prevalence of such behaviors among mothers of normal-weight children, but aligns with the results of Gerards et al.^22^

 Following the identification of the primary challenges encountered by mothers, the subsequent step involves examining the factors that influence their perceptions of these issues and their self-efficacy in managing behaviors associated with childhood obesity. Multiple linear regression analyses indicated that maternal education and child BMI significantly predict mothers’ perceptions of obesity-related issues and their confidence in addressing these challenges. Mothers of overweight or obese children reported higher rates of behavioral problems and lower confidence in managing these issues compared to mothers of normal-weight children, consistent with prior studies.^4,22^ This effect size is not only statistically significant but also clinically meaningful, as even modest increases in child BMI can present substantial challenges for parents particularly mothers in managing their children’s health-related behaviors. These findings are consistent with the broader literature on parental self-efficacy, which indicates that parents of children with higher BMI frequently experience heightened difficulties in child-rearing. Such challenges include increased emotional strain, frustration, and a reduced sense of parental competence, largely driven by children’s resistance to adopting healthy dietary habits and engaging in regular physical activity.^27,37^ No associations were found between maternal occupation, age, number of children, or the child’s age and gender and mothers’ perceptions of childhood obesity-related behaviors or their confidence in addressing them. However, mothers with a high school diploma or lower education level perceived more problems related to their children’s obesity-related behaviors, but showed lower self-efficacy in managing these behaviors compared with mothers who had higher levels of education. This disparity may be attributed to greater health literacy and understanding of healthy behaviors among more educated mothers, enabling them to make informed health decisions for their children.^38,39^

 Previous research on the relationship between maternal education and childhood obesity has produced mixed findings. Some studies suggest that higher maternal education is associated with an increased risk of childhood obesity,^40^ whereas others indicate that having college-educated parents may protect against obesity in six-year-old children. ^41^ Furthermore, mothers with higher education levels are more likely to provide higher-quality diets and more nutritious foods, potentially reducing the prevalence of childhood overweight.^42^ Cross-sectional studies also demonstrate that children of mothers with lower education levels are at greater risk of unhealthy diets, reduced physical activity, and excessive screen time.^43-50^

 The regression model accounted for 11.6% of the variance in maternal problem perception scores and 13.3% of the variance in self-efficacy scores, largely explained by demographic characteristics and child BMI. While these figures indicate statistically meaningful associations, the modest explanatory power underscores the likelihood that other unmeasured factors—such as cultural norms, psychosocial stressors, and parenting beliefs—play a more substantial role in shaping mothers’ perceptions and confidence in managing childhood obesity. These findings highlight the importance of future research adopting multidimensional frameworks that capture the interplay of individual, familial, and sociocultural determinants to more fully elucidate the complexities of parental self-efficacy in this context.

 This study highlights divergent maternal perspectives on behaviors associated with obesity and on self-efficacy in managing these behaviors among children with and without overweight or obesity. However, several limitations should be considered. First, BMI data were obtained from the Iranian Electronic Health Record System (SIB) rather than through direct measurement. Second, the study may be influenced by self-report bias, as responses could reflect social desirability or recall errors. Third, only mothers were included as primary caregivers, which may reflect societal norms and traditional gender roles. Given evolving family dynamics and increasing paternal involvement in childcare, it remains unclear whether fathers share similar perceptions regarding children’s obesity-related behaviors. Finally, the cross-sectional design of the study precludes establishing causal relationships between children’s behaviors, maternal perceptions of obesity-related challenges, and maternal self-efficacy in addressing these issues.

## Conclusion

 This study revealed that a substantial proportion of participants engaged in behaviors that contribute to childhood obesity, with nearly one-third reporting low self-efficacy in addressing these challenges. Mothers most frequently cited sedentary lifestyles as the primary concern. Notably, those with overweight or obese children and lower educational attainment exhibited particularly low confidence in managing this issue. To design effective interventions, future qualitative research should explore the underlying factors that diminish maternal self-efficacy. In light of the growing prevalence of screen time and its established association with childhood obesity especially when coupled with low maternal efficacy in regulating media use this area represents a critical priority for child health promotion. Accordingly, training workshops should be developed to enhance mothers’ capabilities in managing their children’s screen time in alignment with WHO guidelines. These programs should integrate behavioral strategies with technological tools (e.g., screen monitoring apps) to bridge the gap between awareness and implementation, particularly among mothers with limited educational backgrounds. Furthermore, implementing comprehensive, multidimensional interventions encompassing educational, technological, and culturally sensitive components focused on maternal empowerment, represents a strategic approach to improving child health outcomes. Such efforts are particularly vital within national public health agendas targeting vulnerable populations.

## Competing Interests

 The authors declare that they have no conflict of interest.

## Data Availability Statement

 Data supporting this research are available from the corresponding author upon reasonable request

## Ethical Approval

 The Ethics Committee of Zanjan University of Medical Sciences approved this research (Approval ID: IR.ZUMS.REC.1401.335). After obtaining permission from university officials and Vice-Chancellor for Health, and securing informed consent from each participant, we assured them that participation was voluntary and non-participation would not affect healthcare services. Participants were also informed and briefed about the research and questionnaire.
